# Stability and dynamics of dendritic spines in macaque prefrontal cortex

**DOI:** 10.1093/nsr/nwac125

**Published:** 2022-06-27

**Authors:** Ming Chen, Junqian Qi, Muming Poo, Yang Yang

**Affiliations:** Institute of Neuroscience, State Key Laboratory of Neuroscience, Key Laboratory of Primate Neurobiology, Center for Excellence in Brain Science and Intelligence Technology, Chinese Academy of Sciences, Shanghai 200031, China; Institute of Neuroscience, State Key Laboratory of Neuroscience, Key Laboratory of Primate Neurobiology, Center for Excellence in Brain Science and Intelligence Technology, Chinese Academy of Sciences, Shanghai 200031, China; School of Life Science and Technology, ShanghaiTech University, Shanghai 201210, China; University of Chinese Academy of Sciences, Beijing 100049, China; Institute of Neuroscience, State Key Laboratory of Neuroscience, Key Laboratory of Primate Neurobiology, Center for Excellence in Brain Science and Intelligence Technology, Chinese Academy of Sciences, Shanghai 200031, China; School of Life Science and Technology, ShanghaiTech University, Shanghai 201210, China; University of Chinese Academy of Sciences, Beijing 100049, China; Shanghai Center for Brain Science and Brain-Inspired Intelligence Technology, Lingang Laboratory, Shanghai 200031, China; School of Life Science and Technology, ShanghaiTech University, Shanghai 201210, China

**Keywords:** long-term two-photon imaging, macaque monkey, spine plasticity, dendritic spine distribution, regulation of spine dynamics, dorsal lateral prefrontal cortex

## Abstract

Formation and elimination of synapses reflect structural plasticity of neuronal connectivity. Here we performed high-resolution two-photon imaging of dendritic spines in the prefrontal cortex of four macaque monkeys and found that spines were in general highly stable, with low percentages undergoing synaptic turnover. By observing the same spines at weekly intervals, we found that newly formed spines were more susceptible to elimination, with only 40% persisting over a period of months. Analyses of spatial distribution of large numbers of spines revealed that spine distribution was neither uniform nor random, favoring inter-spine distances of 2–4 μm. Furthermore, spine formation and elimination occurred more often in low- and high-density dendritic segments, respectively, and preferentially within a hot zone of ∼4 μm from existing spines. Our results demonstrate long-term stability and spatially regulated spine dynamics in the macaque cortex and provide a structural basis for understanding neural circuit plasticity in the primate brain.

## Teaser

Reported high resolution long-term observation of dendritic spines in macaque, demonstrated the spine dynamics of L5 dlPFC neurons in puberty and adult monkeys and the spatial rule of spine distribution.

## INTRODUCTION

Synapses are considered the primary sites for memory storage in the brain [1] and dendritic spines are the postsynaptic structures of excitatory synapses [2,3]. There are two aspects of spine dynamics: the temporal dynamics, i.e. the dynamic turnover of spines over time; and the spatial dynamics, i.e. the dependence of dendritic locations of spine formation and elimination relative to existing spines. Using *in vivo* two-photon microscopy, the temporal dynamics of spine formation and elimination have been extensively studied in rodents [4–8], demonstrating experience and learning associated spine structural changes in both sensory and high-order cortices. Compared to rodents, primates have a much longer lifespan. It has been suggested that the absolute age of an animal, rather than the relative age, plays an important role in determining neuronal properties, such as the extent of adult neurogenesis in the hippocampus [9,10]. Given the comprehensive studies in temporal spine dynamics in rodents, it is important to examine whether similar spine dynamics occur in the young and adult non-human primate brains.

Non-human primates are close to humans in their brain structure, developmental and aging processes, as well as having higher cognitive functions, and are thus valuable models for studying spine dynamics. Although *in vivo* imaging of calcium signals at the soma level has been achieved for the macaque brain [11], high-resolution imaging of dendritic spines is technically challenging due to difficulties in maintaining long-term image quality and stability, and more severe immune responses in macaques than in rodents. Structural imaging at the synaptic level has been reported in macaque monkeys for axonal boutons in the primary visual cortex, spanning 35 days [12,13], but the analysis of bouton dynamics was limited due to the small amount of repeated imaging.

For studies on spatial dynamics of spines, non-uniform distribution of newly formed spines was observed in mouse hippocampal slices after chemically induced long-term synaptic potentiation, with new spines emerging at low-spine-density regions [14]. Motor learning induced clustering of newly formed spines and clustered spines are more likely to persist after training [7]. Thus, the spatial distribution and the survival of newly formed spines in rodents could be affected by the location of pre-existing spines and spine remodeling could lead to redistribution of spines that reflect rewiring of synaptic connections relevant to learning and memory. Understanding the rules governing spatial regulation of synapse turnover requires further quantitative analysis on the location of spine formation and elimination.

In this study, by designing a custom-made large cranial window (diameter 15 mm) and tailoring surgical procedures, we achieved long-term high-quality *in vivo* imaging of dendritic spines in the macaque prefrontal cortex over months. The Layer 5 (L5) pyramidal neurons were labeled using adeno-associated virus expressing green fluorescent protein and the apical dendrites were repeatedly imaged at intervals ranging from hours to months. We measured the spine-turnover dynamics in four macaque monkeys and found that spines are in general stable over long periods, with few formation and elimination events. The newly formed spines were more susceptible to elimination but their survival rate reached a plateau after 3–4 weeks. Furthermore, we delineated the rules for the spatial distribution of stable, newly formed and eliminated spines. Our results provide a detailed analysis of the spatio-temporal dynamics of synapses in macaque prefrontal cortex and offer insights into the plasticity of synaptic connectivity in the primate brain.

## RESULTS

### Temporal dynamics of dendritic spines in macaque prefrontal cortex

**Figure 1.**
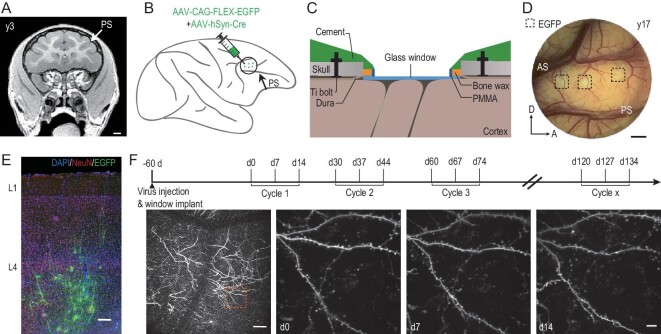
Long-term imaging of dendritic spines in macaque prefrontal cortex. (A) Magnetic resonance structural imaging of the brain of monkey y3 for localizing dorsolateral prefrontal cortex (dlPFC) and determining the sites of viral injection (Layer 5). PS, principal sulcus. Scale bar, 1 mm. (B) Schematic drawing showing the fluorescent labeling of dlPFC neurons by injecting a combination of AAV-flex-EGFP and AAV-syn-cre. Green dots represent multiple injection sites. PS, principal sulcus. (C) Illustration of the custom-made imaging chamber assembly implanted over dlPFC. PMMA, polymethyl methacrylate. (D) Fluorescent image taken through the imaging chamber of monkey y17. Dashed squares represent sites of GFP expression. PS, principal sulcus; AS, arcuate sulcus. Scale bar, 2 mm. (E) Images showing GFP-labeled cells (green) in a brain slice of monkey y13, stained with DAPI (blue) and neuronal marker NeuN (red). L1, Layer 1; L4, Layer 4. Scale bar, 200 μm. (F) Experimental schedule. Two-photon imaging starts 60 days after virus injection, repeating at 7-day intervals for each imaging cycle of three imaging sessions. Bottom, example of two-photon images of monkey y3, the boxed region showing magnified images of same dendritic segments taken on d0, d7 and d14. Scale bars, 100 μm (left) and 10 μm (right).

To examine the structural dynamics of synapses in the macaque cerebral cortex *in vivo*, we used two-photon fluorescence microscopy to image apical dendrites of L5 pyramidal neurons in the dorsolateral prefrontal cortex (dlPFC). Neurons were fluorescently labeled by injecting a mixture of low-titer AAV2/8-hSyn-Cre and high-titer AAV2/9-CAG-FLEX-EGFP viruses into L5 to ensure sparse labeling and a high expression level, with injection sites predefined using magnetic resonance imaging of the macaque brain under study (Fig. 1A and B). A custom-made chronic cranial window was implanted over the dlPFC on the same day as viral injection (Fig. 1C and Supplementary Fig. 1A). Two-photon imaging of the fluorescently labeled apical dendrites in the dlPFC for four monkeys (3, 5, 13 and 17 years old, termed y3, y5, y13 and y17) were performed 60 days after viral injection to allow sufficient viral expression (Fig. 1D, E and Supplementary Fig. 1B). Each imaging cycle consisted of three imaging sessions at 7-day intervals (Fig. 1F). In three monkeys (y3, y5 and y13), an additional one or two imaging cycles were performed 1–4 months after the first cycle (Fig. 1F and Supplementary Fig. 1C). The four monkeys were categorized into two groups: young (y3 and y5, adolescent stage) and middle-aged (y13 and y17, adulthood) [15,16].

**Figure 2.**
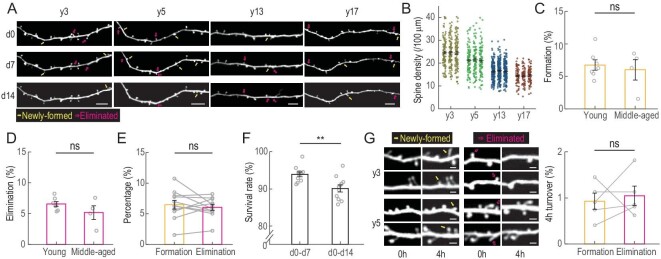
Temporal dynamics of dendritic spines in macaque prefrontal cortex. (A) Example images of the same apical dendrites in dlPFC of four monkeys (y3, y5, y13, y17) obtained by repeated two-photon imaging on d0, d7 and d14. Yellow arrows, newly formed spines; magenta arrows, eliminated spines. Scale bars, 10 μm. (B) Spine density of four monkeys of different ages. Each data point represents one dendritic segment. (C and D). Percentages of spines that were newly formed (C) or eliminated (D) within the 7-day imaging interval in dlPFC of young (y3 and y5) and middle-aged (y13 and y17) monkeys during the first imaging cycle. (E) Averaged 7-day spine formation and elimination rates shown by connected open circles for all injection sites from all four monkeys. (F) Percentages of spines that persisted after 7 and 14 days. (G) Spine dynamics within 4-hour imaging intervals of young monkeys. Left, example images of the same apical dendrites in dlPFC of y3 and y5 obtained by repeated imaging. Yellow arrows, newly formed spines; magenta arrows, eliminated spines. Right, percentages of newly formed (yellow) and eliminated (magenta) spines. Scale bar, 2 μm. Data are presented as mean ± SEM. Each circle represents data from one virus injection site, *n* = 7 sites for young and *n* = 4 for middle-aged monkeys, *n* = 5 sites for (G). Student's *t*-test was used in (C), (D) and (F), paired Student's *t*-test in (E) and (G); ns, not significant; *P < 0.05; **P < 0.01; ***P < 0.001.

Compared to rats (∼300 g) and mice (∼25 g), macaques have much larger body sizes (∼5–15 kg). This caused a problem for *in vivo* spine imaging: the heart beat and breathing can cause vertical movements of the macaque brain tissue of ≤5 μm, which is much larger than the typical size (∼1 μm) of spines. To achieve stable high-resolution imaging of spines, we designed a biocompatible chronic window that imposed a moderate pressure over the exposed cortex and used a high scanning speed of 30 frames per second with multiple repeats to ensure coverage along the *z*-axis. Raw images were post-processed to obtain high-resolution 3D image stacks (Supplementary Fig. 2A and Supplementary Video). Thus, dendritic spines of different shapes and sizes can be imaged at a high signal-to-noise ratio and the same dendrites can be repeatedly imaged over a period of ≥2 weeks, with formation and elimination of spines observed (Fig. 2A). The spine density progressively declines with age (Fig. 2B; total spine number: 7764, 6217, 5408 and 3466 for y3, y5, y13 and y17, respectively. Supplementary Table 1), consistently with previous histological findings in macaques [17–19]. The percentages of spine formation and elimination were calculated by comparing the images obtained on Day 7 (d7) with those obtained on d0, and d14 with d7. The young (y3 and y5) and the middle-aged (y13 and y17) monkeys showed comparable rates of spine formation and elimination (Fig. 2C, D and Supplementary Fig. 2D–F; detailed statistics in Supplementary Table 1). Notably, formation and elimination were balanced for all monkeys (6.6% ± 0.7% vs. 6.0% ± 0.5%, paired Student's *t*-test, *P* = 0.47; Fig. 2E), consistently with the finding that the spine density in these monkeys remained unchanged over the 2-week periods of imaging (Fig. 2B).

A notable feature for monkeys of all ages was the high stability of spines in general. Among all spines observed on d0, >90% were still present on d7 and 85% on d14 (Fig. 2F; Student's *t*-test, *P* = 0.003, statistics in Supplementary Table 1). To further investigate whether spines undergo fast reversible turnover over a shorter period, we performed short-term repeated imaging in y3 and y5 at an interval of 4 hours. We found the probability of formation and elimination within 4 hours to be ∼1% (spine number = 1037 and 1670 in y3 and y5, respectively; 0.9% ± 0.2% vs. 1.1% ± 0.2%, paired Student's *t*-test, *P* = 0.69; Fig. 2G). This 4-hour turnover rate is rather high, considering the average formation/elimination rate observed at the 7-day interval is only ∼6%. This implies that repeated turnover of spines occurred at the same site within the 7-day interval of long-term imaging described above.

### Preferential elimination of newly formed spines

**Figure 3.**
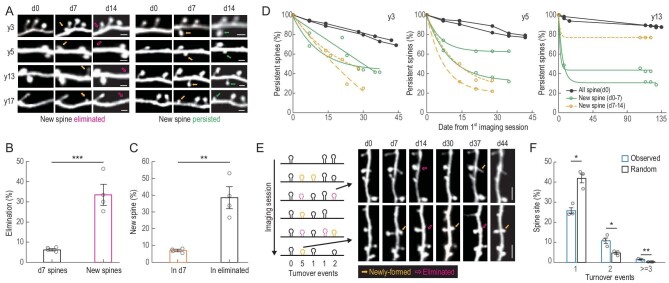
Preferential elimination of newly formed spines. (A) Example images showing newly formed spines observed on d7 (yellow arrows) and then eliminated during d7–d14 (magenta arrows), or persisted until d14 (green arrows). Scale bars, 2 μm. (B) Percentages of spines that were eliminated during d7–d14 among all spines observed on d7 (black) and among newly formed spines observed on d7 (magenta). (C) Percentages of newly formed spines observed on d7 among all spines (orange) and among spines eliminated (black) during d7–d14. (D) Percentages of spines that persisted on subsequent imaging sessions among all spines observed on d0 (black), among newly formed spines observed on d7 (green) and among newly formed spines observed on d14 (yellow), for y3, y5 and y13. (E) Example images showing multiple formation (yellow arrows) and elimination (magenta arrows) events at the same dendritic sites across six imaging sessions. Scale bars, 5 μm. (F) Percentages of spine sites with one, two and at least three spine-turnover events (formation + elimination) for observed (blue) and random simulation (black) across six imaging sessions for y3, y5 and y13. Data are presented as mean ± SEM. Each circle represents data from one monkey. Student's *t*-test was used in (B) and (C), *n* = 4 monkeys; paired Student's *t*-test in (F), *n* = 3 monkeys; ns, non-significant; *P < 0.05; **P < 0.01; ***P < 0.001.

By tracking spines that were newly formed between consecutive imaging sessions (Fig. 3A), we found that these spines were more likely to be eliminated than pre-existing spines. On average, ∼33% of newly formed spines were eliminated during the following week, whereas the percentage of elimination for pre-existing spines over 7 days was only 6% on average (6.2% ± 0.5% vs. 33.5% ± 4.0%, *n* = 4 monkeys, Student's *t*-test, *P* = 0.00096; Fig. 3B). This preferential elimination was also reflected by the high percentages of newly formed spines among the population of eliminated spines (7.1% ± 0.5% vs. 38.5% ± 4.9%, *n* = 4 monkeys, Student's *t*-test, *P* = 0.0014; Fig. 3C).

To analyse the long-term turnover dynamics of pre-existing and newly formed spines, we imaged three monkeys (y3, y5 and y13) for an additional 1–4 months (Fig. 3D). The percentages of pre-existing and newly formed spines that persisted over the imaging course were quantified for each monkey on each imaging day. In y13, 87% of pre-existing spines persisted over 4 months compared to 30%–70% for newly formed spines. Interestingly, the rapid decline of the new spines ceased after 3–4 weeks and the survival rate reached a plateau, indicating that the new spines that had survived the first few weeks became a stable population.

To determine whether spine formation and elimination tended to occur repeatedly at the same sites, we marked the sites of spine elimination and formation along dendrites with 1-μm spatial resolution and tracked the spine-turnover events on these sites over six imaging sessions (Fig. 3E). We found that ∼10% of the spine sites had two turnover events (formation or elimination) and 1% had three (formation–elimination–formation or elimination–formation–elimination) or more. The percentages of the sites with repeated turnover are significantly higher than those predicted by assuming random turnover across all spine sites, suggesting that there are hotspots for spine turnover (*n* = 3 monkeys, paired Student's *t*-test; turnover = 1, 25.8% ± 1.4% vs. 42.0% ± 2.2%, *P* = 0.022; turnover = 2, 10.6% ± 1.4% vs. 4.7% ± 0.6%, *P* = 0.028; turnover = 3, 1.7% ± 0.1% vs. 0.3% ± 0.05%, *P* = 0.0047; Fig. 3F). This is in line with the finding of repeated turnover at the same sites implicated by the comparison of 4-hour versus 7-day turnover rates described earlier.

### Spine dynamics depend on spine morphology

**Figure 4.**
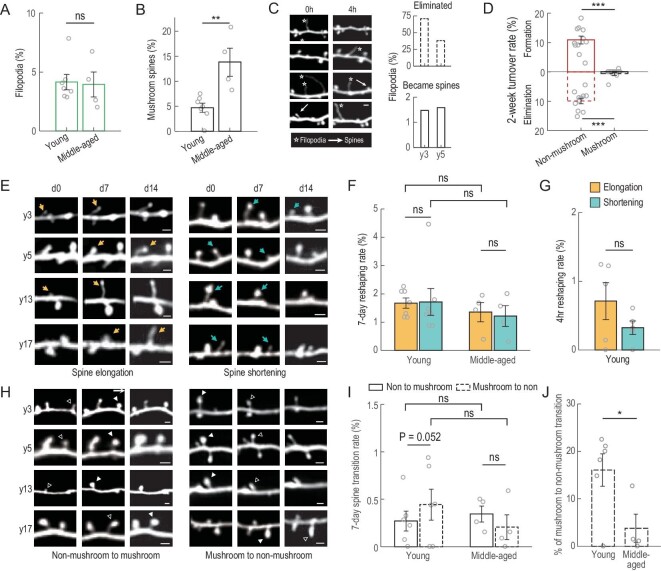
Morphological changes of spines and filopodia. (A) Percentages of filopodia among all protrusions observed in young and middle-aged monkeys. (B) Percentages of mushroom spines observed in young and middle-aged monkeys. (C) Filopodia dynamics within a 4-hour interval in young monkeys. Left: example images showing 4-hour dynamics for filopodia. White arrows, spine; white stars, filopodia. Right: percentages of filopodia that were eliminated or became spines within the 4-hour interval. Scale bars, 2 μm. (D) Formation (solid) and elimination (dashed) rates of non-mushroom (red) and mushroom (black) spines at 2-week intervals. (E) Example images showing elongation (yellow arrow) and shortening (blue arrow) of non-mushroom spines in four monkeys. Scale bars, 2 μm. (F) Percentages of non-mushroom spines undergoing elongation (yellow) and shortening (blue) within 7-day intervals for young and middle-aged monkeys. (G) Percentages of non-mushroom spines undergoing elongation (yellow) and shortening (blue) within 4-hour intervals for young monkeys. (H) Example images showing non-mushroom (open triangle) to mushroom (solid triangle) transitioning (left), and the reverse (right) in four monkeys. Scale bars, 2 μm. (I) Non-mushroom to mushroom spine transition (percentage of all spines, solid bars) and mushroom to non-mushroom (percentage of all spines, dashed bars) within 7-day intervals for young and middle-aged monkeys. (J) Mushroom to non-mushroom spine transition (percentage of all mushroom spines) within 7-day intervals for young and middle-aged monkeys. Data are presented as mean ± SEM except (C). Each circle represents data from one virus injection site, *n* = 7 sites for young and *n* = 4 for middle-aged monkeys; *n* = 5 sites for (G); *n* = 6 sites for young monkeys in (D) (mushroom), (I) and (J). Student's *t*-test was used; ns, non-significant; *P < 0.05; **P < 0.01; ***P < 0.001.

The functional properties of spines are largely determined by the spine morphology [20,21]. We categorized the dendritic protrusions into three subtypes: (i) filopodia, the long thin protrusions without distinctive spine heads; (ii) mushroom spines, with mushroom-shaped spine heads and a thin spine neck; and (iii) non-mushroom spines, including thin and stubby spines, both lacking a distinctive spine head and neck (Supplementary Fig. 3A and B). Filopodia are often considered as the precursors of spines and mushroom spines the mature spines. We found that the proportions of filopodia are ∼4% in both young and middle-aged monkeys, but that of mushroom spines is higher in the middle-aged monkeys than young monkeys (Fig. 4A and B; further results and details in Supplementary Table 2), indicating preferential maintenance of mushroom spines. We also examined short-term dynamics of filopodia in y3 and y5 monkeys and found that <2% became spines within 4 hours (Fig. 4C). Their high dynamics and low probability of turning into spines are consistent with their exploratory functions in spine formation. By contrast, mushroom spines are extremely stable, as shown by their lower rates of formation and elimination over observation periods of 2 weeks and 4 months (Supplementary Fig. 3E), and also as compared to those of non-mushroom spines (Fig. 4D). Newly formed spines are essentially all non-mushroom spines (953/955, four monkeys), corroborating with their higher level of plasticity compared to mushroom spines. This result is different from what had been reported in mice [4,22], in which newly formed spines include both mushroom and non-mushroom spines.

To evaluate the morphological changes of spines, we used two criteria: morphological reshaping including spine elongation and shortening, and subtype transitioning between mushroom and non-mushroom spines. We observed morphological reshaping mainly in non-mushroom spines, as defined by ≥15% elongation or shortening in spine length, with ∼0.5% and ∼1.5% reshaping rates at 4-hour and 7-day imaging intervals (Fig. 4E–G and Supplementary Fig. 3C; further results and details in Supplementary Tables 3 and 7), suggesting wiring modifications of postsynaptic compartments in the monkey brain. Subtype transitioning, including mushroom to non-mushroom and vice versa, were defined as shape change accompanied by ≥30% swelling or shrinkage in spine size at the 7-day interval (Fig. 4H–J and Supplementary Fig. 3D). The percentages of mushroom to non-mushroom transitions in young monkeys are higher than those in middle-aged monkeys, suggesting a lower level of mushroom spine stability in young monkeys (Fig. 4J; further results and details in Supplementary Table 3).

### Quantitative spatial analysis reveals topographic rules for spine distribution

**Figure 5.**
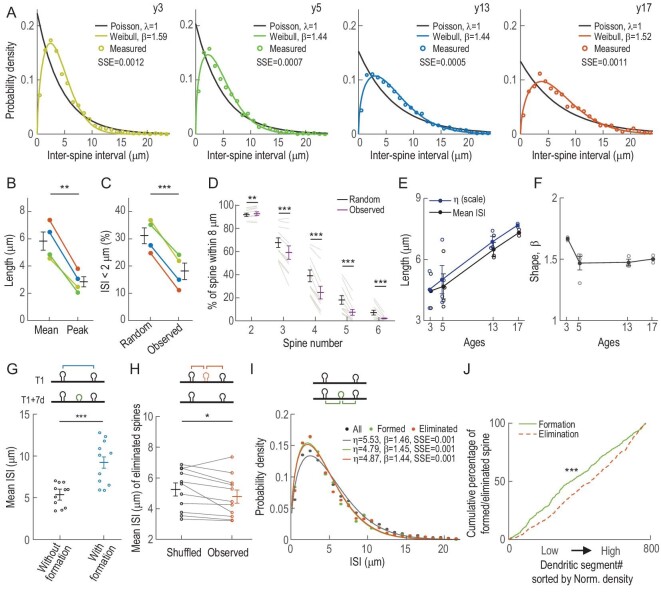
Quantitative spatial analysis reveals topographic rules for spine distribution and spatial dynamics. (A) Probability density function of inter-spine intervals (ISIs) observed in four monkeys. Colored open circles: measured ISIs; colored lines: Weibull fitted distributions; black solid lines: Poisson distributions (*λ* = 1) with the same mean ISIs. (B) Mean and peak ISIs of the best-fit Weibull distributions. (C) Percentages of ISIs of <2 μm for assumed randomly distributed (Poisson distributions, *λ* = 1) and observed ISIs. (D) Percentages of spine clusters with two to approximately six spines within an 8-μm dendritic distance for assumed randomly distributed (Poisson distributions, *λ* = 1) and observed ISIs. (E) Scale parameters (*η*) of the best-fit Weibull distributions for four monkeys across ages. Each circle represents data from one injection site. (F) Shape parameters (*β*) of the best-fit Weibull distributions for four monkeys across ages. Each circle represents data from one injection site. (G) Mean ISIs without (black) and with (blue) new spine insertions in between. (H) Averaged ISIs of shuffled (black) and observed (orange) eliminated spines relative to their nearest neighbors, data from four monkeys. (I) Probability density function of ISIs for all (black), newly formed (green) and eliminated (red) spines relative to their nearest neighbors, data from four monkeys. (J) Cumulative percentages of newly formed (green) and eliminated (red) spines plotted against dendritic segments with different spine densities (sorted from low to high level), data from four monkeys. SSE, sum of squared errors. Data are presented as mean ± SEM. Paired Student's *t*-test in (B) and (C), *n* = 4 monkeys; paired Student's *t*-test in (D), (G) and (H), *n* = 11 virus injection sites; Kolmogorov–Smirnov test in (J); ns, non-significant; *P < 0.05; **P < 0.01; ***P < 0.001.

In addition to synaptic morphology and efficacy, the dendritic location of a spine is critical for determining its contribution to neuronal activity during dendritic integration. For example, the spatial clustering of spines can alter the integration efficiency of synaptic inputs [23–25]. To explore the spatial distribution of spines, we measured the inter-spine intervals (ISIs) between adjacent spines for all spines observed in this study (>1500 each in y3, y5 and y13; >1000 in y17). The distribution of ISIs did not conform to that expected for Poisson distribution (λ = 1, shown as exponential curves), but rather exhibited distinct peaks at 2.5, 2.1, 3.1 and 3.8 μm, for y3, y5, y13 and y17, respectively (Fig. 5A). These peaks were substantially shorter than the mean ISIs (4.6, 4.9, 6.5 and 7.4 μm, respectively; 5.8 ± 0.6 vs. 2.9 ± 0.3 μm; *n* = 4 monkeys, paired Student's *t*-test, *P* = 0.0031; Fig. 5B), suggesting spatial clustering of spines at ∼2–4 μm across all age groups. Notably, at <2 μm, the frequency of ISIs was significantly lower than that expected for the Poisson distribution, indicating that although spines tended to cluster, each spine tended to exclude the presence of other spines within 2 μm (31.1% ± 2.5% vs. 18.1% ± 2.6%; *n* = 4 monkeys, paired Student's *t*-test, *P* = 0.00 057; Fig. 5A and C). In addition, spine clustering was more likely to occur in pairs than triplets or quadruplets; the occurrence of two spines within 8 μm was more frequent than predicted by Poisson distribution, but clustering of three spines or more occurred significantly less often, suggesting that the number of spines in close vicinity (8 μm) was limited (Fig. 5D; further results and details in Supplementary Table 5).

Further analysis revealed that the probability (*P*) of observing certain ISIs (*x*) for each monkey fitted well with a right-skewed Weibull distribution (Fig. 5A):

(1) \begin{equation*} p(x) = \frac{\beta }{\eta }{\left( {\frac{{\rm{x}}}{\eta }} \right)}^{\beta - 1}{\exp }^{ - {{\left( {\frac{x}{\eta }} \right)}}^\beta }, \end{equation*}

where *η* represents the scale parameter and *β* represents the shape parameter. In this fitting, *η* is proportional to the mean ISI observed for each monkey (Supplementary Fig. 4A) and is inversely correlated with the spine density. Thus, *η* values were higher in older monkeys, consistently with the age-dependent decline in spine density (Fig. 2B and E). The shape parameter *β* was found to be largely age-independent (1.67, 1.47 1.47 and 1.50 for y3, y5, y13 and y17, respectively; Fig. 5F). The probability of spine occurrence is lower than that predicted by random insertion at locations near the existing spines, but higher at locations away from the existing spines, and peaks at the midpoint of two adjacent existing spines (Supplementary Fig. 4B).

### Pre-existing spine topography determines locations of spine formation and elimination

To investigate whether the right-skewed distribution of ISIs resulted from spatial preference of spine formation and elimination, we measured the distances of newly formed and eliminated spines relative to their adjacent pre-existing spines and analysed the distribution of those distances. We found that in all monkeys, the average distances of two adjacent spines between which a new spine was later inserted were significantly larger than those without spine insertions (5.4 ± 0.4 vs. 9.2 ± 0.8 μm, *n* = 11 injection sites, paired Student's *t*-test, *P* = 1.87E-06; Fig. 5G). This was demonstrated by the cumulative distributions of ISIs with vs. without spine insertions (Supplementary Fig. 4C). Thus, new spines tended to form at dendritic regions where the density of pre-existing spines was lower. For the dendritic location of spine elimination, the average ISIs between eliminated and pre-existing spines were significantly smaller than those ISIs of shuffled eliminated spines, indicating that spines located at dendritic areas of higher spine density were more likely to be eliminated (5.1 ± 0.4 vs. 4.8 ± 0.4 μm, paired Student's *t*-test, *P* = 0.039, *n* = 11 injection sites; Fig. 5H). We further investigated the spatial relationship between the new spines/the eliminated spines and their adjacent pre-existing spines. Again, the ISIs for newly formed and eliminated spines also conformed to the Weibull distribution, with *η* and *β* values comparable to each other (Fig. 5I and Supplementary Fig. 4D; further results and details in Supplementary Tables 6 and 9).

Direct examination of the spine formation and elimination rates for dendritic segments (with length >50 μm) with varying spine densities showed that the low-density segments had higher formation rates and high-density segments had higher elimination rates. This result was demonstrated by the significant difference between the cumulative probabilities of formation and elimination rates, plotted against dendritic segments sorted by normalized spine densities of all four monkeys (Kolmogorov–Smirnov test, *P* = 1.66E-28; Fig. 5J and Supplementary Fig. 4E). Such density-dependent turnover would even out spine distribution with time. Thus, although there is a tendency for spines to cluster at closer distances, there is a global spatial regulation of spine insertion and elimination that promotes more even distribution of spines along the dendrites.

## DISCUSSION

By performing long-term *in vivo* imaging of dendritic spines in the prefrontal cortex of four macaque monkeys, we found that spines were highly stable, especially the mushroom-shaped spines, while newly formed spines were rarely mushroom-shaped and prone to elimination (Fig. 3). Spatial analyses of spine locations revealed that the intervals of adjacent spines, as well as the intervals of newly formed and eliminated spines to their nearest pre-existing spines, conformed to non-random Weibull distribution. Formation and elimination obeyed different rules: spine formation and spine elimination occurred more frequently at regions with sparse and dense spines (Fig. 5G and H), respectively, leading to a dispersed distribution of spines over time. Nevertheless, spine formation and elimination both had a tendency to occur at short distances (<4 μm) from pre-existing spines (Fig. 5I). Our results demonstrated the dynamics for spine formation and elimination in macaque monkeys and delineated rules for spatial distribution of spines along the dendrite.

Primates differ substantially from rodents in their brain anatomy and lifespan. Information on the dynamics of synaptic structures in the monkey cortex is important for understanding the synaptic basis of neural plasticity underlying cognitive functions and brain disorders in humans. In our study, the 7-day spine-turnover rate (formation + elimination) for monkey dlPFC L5 neurons was 12.4%, which is lower than that observed in mouse motor cortex (20%, 8 days) and dorsomedial prefrontal cortex (18%, 7 days) [26]. As for long-term turnover, the spine survival rate over 1 month is much higher in monkeys than in mice [27–30]. Mushroom spines, which are often considered as mature spines, are particularly stable in macaques. Essentially all mushroom spines (>99%) in the macaque cortex persisted after the 2 weeks and >97% persisted for as long as 5 months. By comparison, mushroom spines in mice showed significant elimination, up to 4% in the frontal cortex and 7% in the barrel cortex within 7 days [5,26,31]. Such a difference is in line with the retention of long-term memories over the lifespan of the animal—months or years for rodents but decades for primates.

We also found three aspects of spine regulation in non-human primates that are similar to those found in rodents: (i) spine density decreases with age; (ii) mushroom spines are more stable than non-mushroom spines [4–6]; and (iii) newly formed spines are more susceptible to elimination. The reduced spine density with age appears to be a normal process of neurodegeneration. The high stability of mushroom spines is in accordance with the notion that these spines encode long-term memory. Long-term survival of new spines has been investigated in mouse motor cortex and it has been shown that about half of the new spines disappear within 2 days, and the long-term survival rate of new spines plateaus at 20% after ∼2 weeks [29,32]. In comparison, the new-spine survival rate plateaus at 40% after 3–4 weeks in monkeys (Fig. 3D). This higher stability of spines in monkeys may indicate more efficient use of cellular resources for synaptic remodeling underlying memory processes.

In addition to spine temporal dynamics, an important aspect of our work is the analyses of the spatial distribution and spatial dynamics of spines. Our analysis revealed that spines are neither uniformly nor randomly distributed along the dendrites; rather, the ISIs of spines conformed to a non-random Weibull distribution, determined by both density-dependent and density-independent parameters. Such right-skewed ISI distribution had also been observed in pyramidal neurons of M1 and CA1 in rodents [33,34], suggesting cellular constraints on the overall distribution of spines. But this ISI distribution does not appear to occur in somatostatin-expressing Martinotti cells in the mouse cortex, which follow an exponential distribution [35]. The rule we described here for Layer-5 pyramidal cells thus may not apply to other neuronal types.

By further analysing the dendritic locations of dynamic spines, we noted that some locations were highly active, with repeated spine appearances and disappearances (Fig. 3E and F). This result suggested that some new spine formation and elimination might represent connection and disconnection, respectively, with a pre-existing presynaptic bouton situated near the active location. We also found that formation and elimination happened more often in low- and high-density regions, respectively, implying resource competition for synaptic components at the level of dendritic segments. Regardless of spine density, however, there appeared to be a hot zone for spine turnover at short distances (<4 μm) from existing spines, reminiscent of the phenomena of heterosynaptic plasticity at nearby synapses observed in hippocampal pyramidal cells [1,36–39]. Crosstalk between neighboring spines on the same dendrite within short distances can lower the threshold for synapse-specific long-term potentiation [36,40,41].

As gene editing of non-human primates can now be achieved, monkeys are becoming valuable models for studying human diseases associated with genetic variations [42–44]. Previous histological studies have shown that Alzheimer's disease leads to severe spine loss [45,46], but it is unclear whether the loss is due to decreased formation, increased elimination or reduced stability of synapses. There is evidence that autism spectrum disorders are associated with increased spine density [47,48], whereas schizophrenia is associated with reduced spine density [49,50], but whether such spine dysregulation is due to abnormal spine formation or pruning remains to be clarified. The genetic causes of dysregulation in various aspects of spine turnover are also largely unknown. Long-term recording of spine dynamics *in vivo* using non-human primate disease models could help to address these issues and to evaluate the efficacy of potential therapeutic approaches. Our results also pave the way for studying the plasticity of synaptic structures associated with higher-order cognitive functions in non-human primates.

## MATERIALS AND METHODS

For detailed materials and methods, please see the Supplementary data.

## Supplementary Material

nwac125_Supplemental_Files
