# Supplementary material for: Stability and dynamics of dendritic spines in macaque prefrontal cortex
Source: Natl Sci Rev. 2022 Jun 27;9(9):nwac125. doi: 10.1093/nsr/nwac125 (PMC9521340; doi:10.1093/nsr/nwac125)
Supplement: nwac125_Supplemental_Files [file nwac125_supplemental_files.zip › Supplementary_data-Figures_and_Tables.pdf]

Supplementary Figure 1

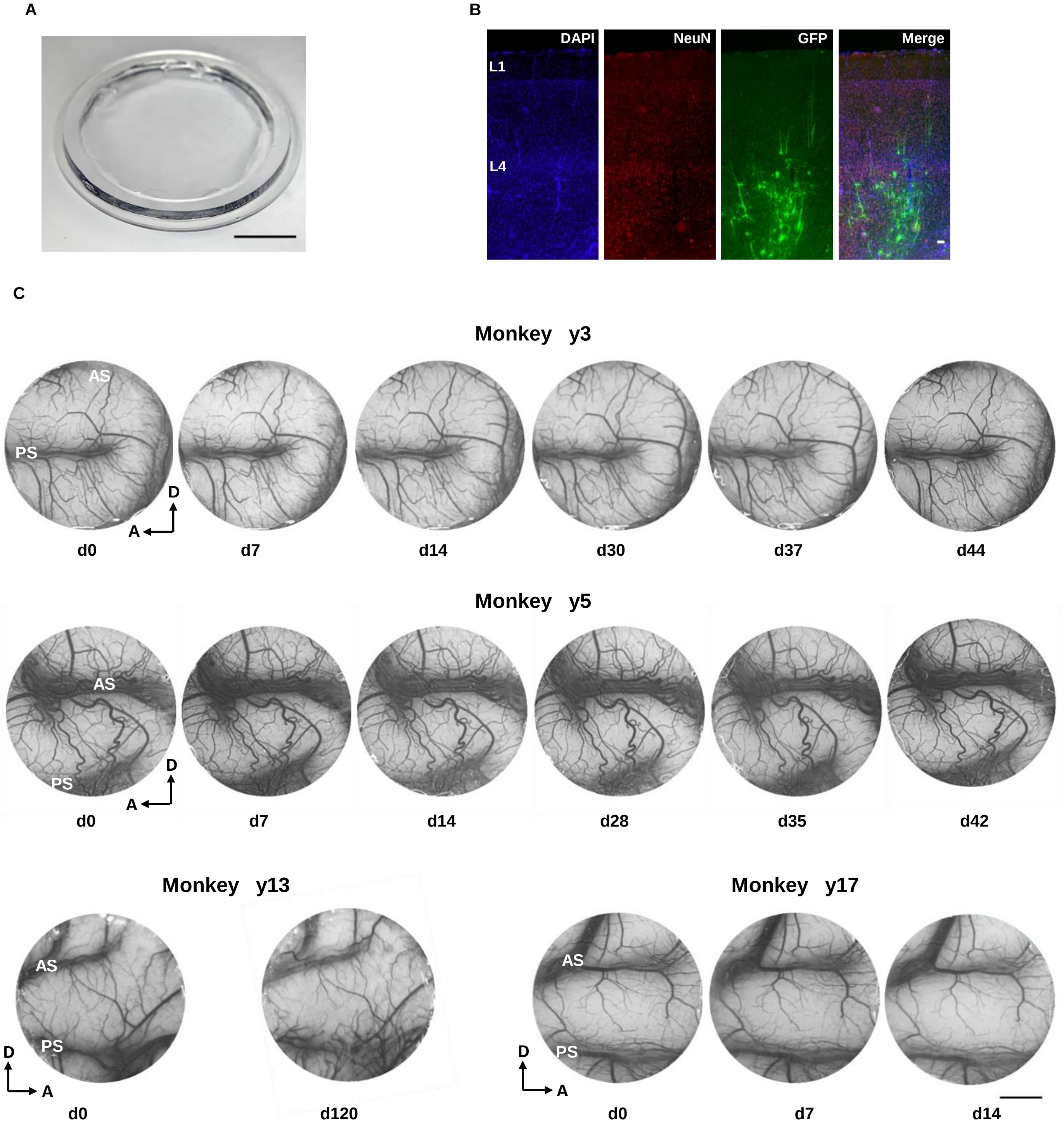

Supplementary Figure 1. Consistency of chronic cranial window on 4 monkeys.

A. Custom-made glass imaging chamber. Scale bar, 5 mm.

B. Images showing GFP-labeled cells (green) in a brain slice of monkey y13, stained with DAPI (blue) and neuronal marker NeuN (red). Scale bar, 100 μm.

C. Images showing the chronic cranial window over the dlPFC of 4 monkeys on different days of imaging. PS, principal sulcus; AS, arcuate sulcus. Scale bar, 5 mm.

Supplementary Figure 2

A

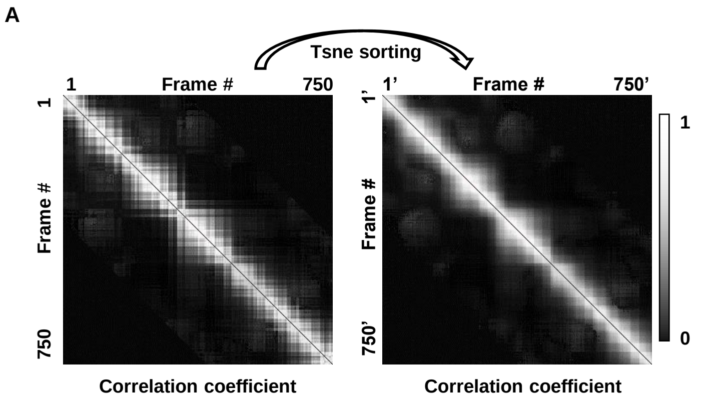

C

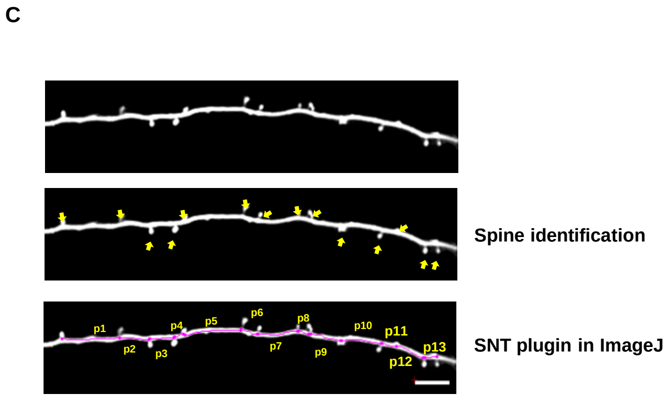

B

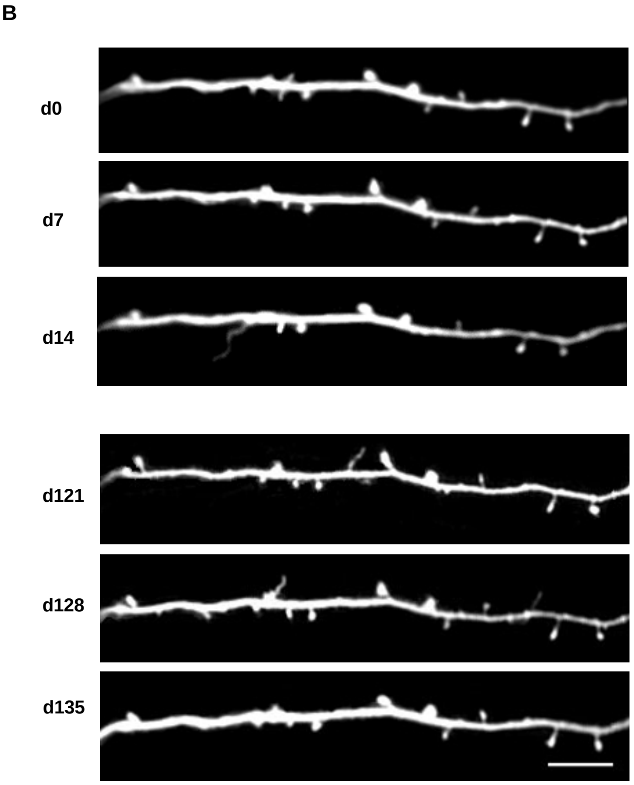

Path	1	2	3	4	5	6	7	8	9	10	11	12	13
Length (μm)	16.707	8.482	6.531	2.723	16.437	5.295	12.096	3.371	9.382	11.099	4.937	8.943	3.744
Sum		Spines		Density(/100μm)									
109.749	14			12.76									

	Spine formation	Spine elimination
T0<br>T0+7d, or T0+14d	ISI_1<br>ISI_a ISI_b	ISI_2 ISI_3
ISI calculate formula	First acquire ISI_a, ISI_b .and ISI_1<br>then determined ISI_c , ISI_d as follows,<br>$ISI_c = ISI_a /(ISI_a + ISI_b) \times ISI_1$<br>$ISI_d = ISI_b /(ISI_a + ISI_b) \times ISI_1$<br>so that $ISI_c + ISI_d = ISI_1$:<br>ISI_c ISI_d	$ISIe = ISI_2 + ISI_3$<br>ISIe

D

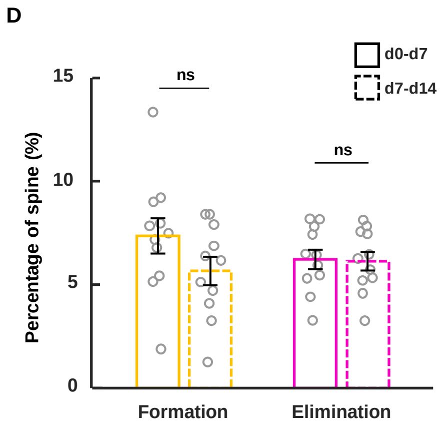

E

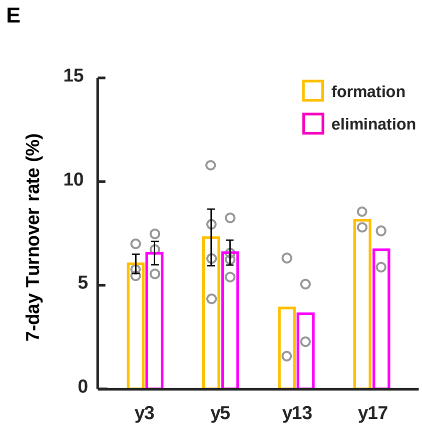

F

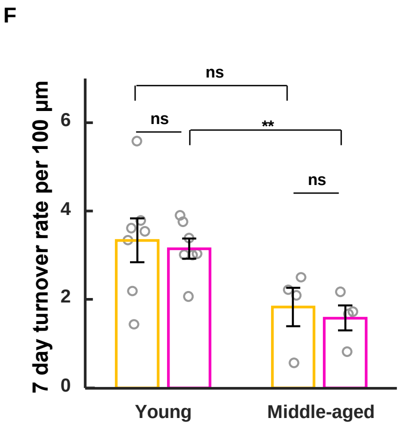

Supplementary Figure 2. Acquisition and consistency of long-term high-resolution two-photon imaging of L5 dendrite spines and spine identification, inter-spine interval measurement and spine turnover comparison.

A. Imaged alignment of z-axis by t-sne (t-distributed stochastic neighbor embedding) sorting.

B. Example images of the same apical dendrite in dlPFC of y13 taken on d0, d7, d14, d120, d127 and d134. Scale bar, 10 μm.

C. ISI measurement for newly formed and eliminated spines.

D. Comparison of 7-day turnover rate between d0-d7 (solid) and d7-d14 (dashed) for spine formation (yellow) and elimination (magenta) for young and middle-aged monkeys.

E. Percentages of spines formation (yellow) and elimination (yellow) within the 7-day intervals (by comparison of d7 vs. d0, and d14 vs. d7) for 4 monkeys.

F. 7-Day spine formation (yellow) and elimination (magenta) rates per 100 μm for young and middle-aged monkeys.

Data was presented as mean ± SEM. Each circle represents data from one virus injection site, n = 7 sites in young and n = 4 sites in middle-aged monkeys. Student's t-test in **D**, paired Student's t-test in **F**; ns, non-significant, *P < 0.05; **P < 0.01; ***P < 0.001.

Supplementary Figure 3

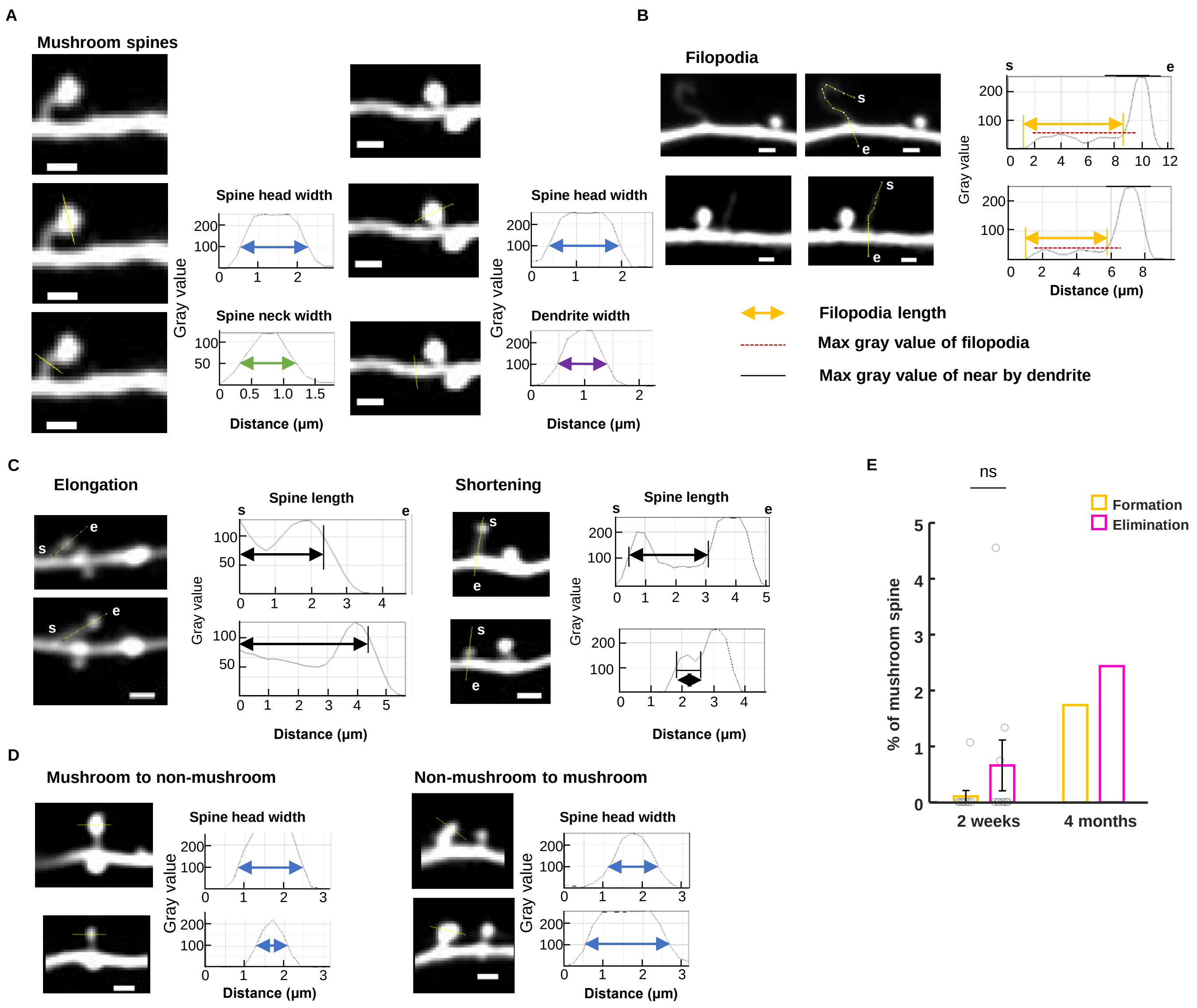

Supplementary Figure 3. Spine type and morphology change identification.

A. Mushroom spine categorization using three measurements: spine head width, spine neck width and dendrite width, quantified by gray value measured from Plot Profile (ImageJ), with clearly visible (left) and unidentifiable (right) spine necks, respectively.

B. Filopodia identification by gray value or protrusion length quantified by Plot Profile.

C. Reshaping for non-mushroom spines defined by spine length change, quantified by Plot Profile.

D. Transitioning defined by spine length change, quantified by Plot Profile. Left, mushroom spine to non-mushroom spine; right, non-mushroom spine to mushroom spine.

E. Mushroom spine formation (yellow) and elimination (magenta) rates within the 7-day intervals for all monkeys and within 4-month intervals for y13.

Scale bars are 2 µm. Data in **E** was presented as mean ± SEM. Each circle represent data from one virus injection site, total 10 sites, 6 for young and 4 for middle-aged monkeys.

Supplementary Figure 4

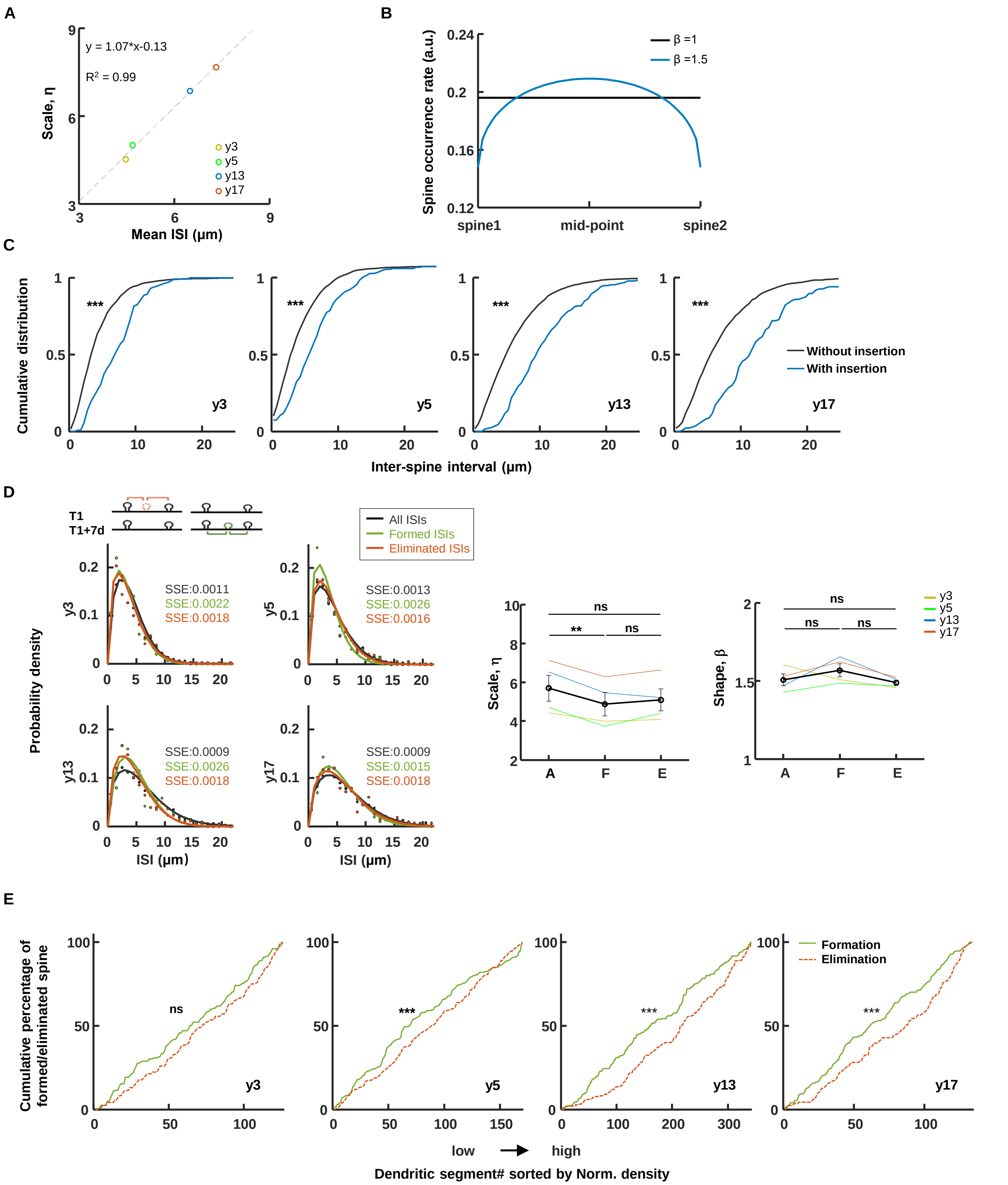

Supplementary Figure 4. Quantitative spatial analysis for inter-spine intervals.

A. Correlation between mean ISI and the best-fitted scale parameter (η) for 4 monkeys.

B. Simulation of spine occurrence rate at locations between 2 existing spines, for shape parameters $\beta = 1$ (black line, random distribution) and $\beta = 1.5$ (blue line, best-fit for our data) of the Weibull distribution.

C. Cumulative distributions of all ISIs (black) and ISIs with spine insertion (blue) for 4 monkeys.

D. Left, probability density function of inter-spine intervals (ISIs) for all (black), newly-formed (green) and eliminated (red) spines in 4 monkeys. Right, scale and shape parameters of best-fit Weibull distributions for all (A), newly formed (F) and eliminated (E) spine for 4 monkeys (mean ± SEM). SSE, sum of squared errors.

E. Cumulative percentages of newly formed (green) and eliminated (red) spines plotted against dendritic segments with different spine densities (sorted from low to high level) in 4 monkeys.

Paired Student's t-test in **D**, n = 4 monkeys; Kolmogorov-Smirnov test in **C** and **E**; ns, non-significant, $*P < 0.05$; $**P < 0.01$; $***P < 0.001$.

Supplementary Table 1. Summary of statistical analyses

Fig	Numeric data and statistical p value					
		dendritic segments	density (/100 um)			length>50μm
**2B**	y3	99	24.59 ± 0.64	24.50 ± 0.60	24.52 ± 0.66	
	y5	86	21.14 ± 0.62	21.50 ± 0.65	21.18 ± 0.69	
	y13	104	17.05 ± 0.36	17.43 ± 0.37	17.21 ± 0.35	
	y17	66	14.30 ± 0.43	14.34 ± 0.42	14.53 ± 0.42	

Fig			injection sites							mean	s.e.m
**2C-D**	formation (%)	young	5.72	6.96	5.41	6.26	7.91	10.75	4.31	6.76	0.79
		middled-aged	1.54	6.28	7.76	8.51				6.02	1.56
	elimination(%)	young	6.69	5.51	7.45	5.36	8.22	6.51	6.20	6.56	0.38
		middled aged	2.23	5.02	7.58	5.84				5.17	1.12
	young vs. middle-aged		formation	P = 0.65							
	Student's t-test		elimination	P = 0.18							

Fig		injection sites											mean	s.e.m
**2E**	formation (%)	5.72	6.96	5.41	6.26	7.91	10.75	4.31	1.54	6.28	7.76	8.51	6.57	0.69
	elimination (%)	6.69	5.51	7.45	5.36	8.22	6.51	6.20	2.23	5.02	7.58	5.84	5.99	0.46
	paired Student's t-test									P = 0.47				

Fig	survival rate (%)										
**2F**	d0-d7	91.2	94.1	92.9	94.6	91.6	94.6	94.0	98.8	95.7	92.3	94.6
	d0-d14	88.0	91.4	87.2	91.9	85.8	91.0	89.7	96.3	93.5	86.6	90.5
		mean	s.e.m									
	d0-d7	94.0	0.6	d0-d7 vs. d7-d14, Student's t-test								
	d0-d14	90.2	0.9	P = 0.0028, **								

Fig	4h turnover						mean	s.e.m
**2G**	formation (%)	0.91	1.13	1.45	0.41	0.74	0.93	0.16
	elimination (%)	1.83	1.13	0.62	0.82	0.85	1.05	0.19
	Student's t-test	P = 0.67, ns						

Fig	elimination rate (%)	y3	y5	y13	y17	mean	s.e.m
**3B**	d7 spines	5.91	6.83	4.82	7.11	6.17	0.45
	new spines	29.71	33.14	46.08	25.00	33.48	3.91
	Student's t-test	P = 0.00096, ***					

Fig	new spines ratio (%)	y3	y5	y13	y17	mean	s.e.m
**3C**	in d7 spines	6.76	8.21	5.59	7.63	7.05	0.49
	in eliminated spines (d14)	33.99	39.86	53.41	26.83	38.52	4.88
	Student's t-test	P = 0.0014, **					

Supplementary Table 2. Summary of statistical analyses

Fig									
**Numeric data and statistical p value**									
**3D**	y3 spine survival rate	all spine		d0	d7	d14	d28/d30	d35/d37	d42/d44
			roi1	321	0.94(303)	0.91(293)	0.83(266)	0.78(251)	0.74(238)
			roi2	598	0.94(561)	0.89(531)	0.80(477)	0.73(436)	0.69(417)
		d0-7 new		d0	d7	d21/d23	d28/30	d35/d37	
			roi1	65	0.82(53)	0.77(50)	0.45(29)	0.42(27)	
			roi2	41	0.68(28)	0.59(24)	0.44(18)	0.41(17)	
		d7-14 new		d0	d14/d16	d21/d23	d28/30		
			roi1	22	0.64(14)	0.55(12)	0.45(10)		
			roi2	24	0.58(14)	0.29(7)	0.25(6)		
	y5 spine survival rate	all spine		d0	d7	d14	d28	d35	d42
			roi1	435	0.94(409)	0.91(394)	0.82(356)	0.79(342)	0.77(332)
			roi2	186	0.95(177)	0.94(175)	0.89(165)	0.81(151)	0.79(147)
		d0-7 new		d0	d7	d21	d28	d35	
			roi1	27	0.74(20)	0.63(17)	0.63(17)	0.63(17)	
			roi2	22	0.64(14)	0.41(9)	0.36(8)	0.32(7)	
		d7-14 new		d0	d14	d21	d28		
			roi1	41	0.32(13)	0.24(10)	0.22(9)		
			roi2	18	0.50(9)	0.33(6)	0.33(6)		
	y13 spine survival rate	all spine		d0	d7	d14	d114	d121	d128
			roi1	617	0.98(607)		0.89(552)	0.89(548)	0.87(539)
			roi2	614	0.97(593)	0.94(576)	0.89(546)	0.89(544	0.88(538)
		d0-7 new		d0	d107/d7	d114	d121	d128	
			roi1	33	0.45(15)	0.42(14)	0.42(14)		
			roi2	35	0.49(17)	0.31(11)	0.31(11)	0.29(10)	
		d7-14 new		d0	d107	d114	d121		
			roi2	13	0.77(10)	0.77(10)	0.77(10)		

Fig	% of spine sites		turnover events within 6 imaging sessions: 1	2	3
**3F**	observed	y3	29.33	10.73	1.70
		y5	24.19	13.35	1.88
		y13	23.88	7.84	1.37
	Random	y3	44.41	5.25	0.31
		y5	45.01	5.43	0.33
		y13	36.52	3.33	0.15
	observed vs. random paired Student’s t-test		P = 0.022, *	P = 0.028, *	P = 0.0047, **

Fig										mean	s.e.m
**4A-B**	filopodia ratio (%)	young	4.09	7.85	2.97	3.37	3.62	2.87	4.35	4.16	0.60
		middle-aged	2.02	4.38	2.63	6.76				3.95	0.92
	mushroom spine ratio (%)	young	5.90	0.10	7.50	3.57	6.54	4.93	4.53	4.72	0.85
		middle-aged	20.82	15.87	8.25	10.45				13.85	2.44

young vs. middle-aged, Student's t-test

P = 0.86, ns   P = 0.0041, **

Fig	filopodia (n)	t0	eliminated	into spines	maintained
**4C**	y3	68	48	1	19
	y5	63	24	1	38

Supplementary Table 3. Summary of statistical analyses

Fig	Numeric data and statistical p value														
														mean	s.e.m
4D	non-mushroom	formation rate (%)	9.05	11.24	9.10	10.14	14.58	18.27	5.79	2.94	9.69	14.21	14.94	10.91	1.26
		elimination rate (%)	10.76	8.31	13.41	8.25	15.26	8.89	9.76	4.50	6.85	13.82	8.81	9.88	0.92
	mushroom	formation rate (%)	0.00	0.00	0.00	0.00	0.00	0.00	0.00	0.00	1.07	0.00		0.11	0.1
		elimination rate (%)	4.55	1.33	0.00	0.00	0.00	0.00	0.00	0.74	0.00	0.00		0.66	0.43
	non-mushroom vs. mushroom		formation			elimination									
	Student's t-test		P = 2.81E-07, ***			P = 9.11E-08, ***									

Fig	7-d reshaping rate (%)										
										mean	s.e.m
4F	young	elongation	2.08	1.16	2.26	1.68	2.08	1.30	1.17	1.68	0.16
		shortening	1.26	0.87	4.46	1.79	1.46	1.30	0.88	1.72	0.44
	middle-aged	elongation	0.39	1.43	1.94	1.67				1.36	0.29
		shortening	0.31	1.56	2.00	1.00				1.22	0.32
	Student's t-test within each group										
			P = 0.94, ns		P = 0.79, ns						
	Student's t-test between group										
		elongation	P = 0.38, ns								
		shortening	P = 0.49, ns								

Fig	4h reshaping rate (%)								
								mean	s.e.m
4G	young	elongation	0.00	0.14	1.24	1.22	0.96	0.71	0.24
		shortening	0.61	0.28	0.41	0.00	0.32	0.32	0.09
	Student's t-test								
	P = 0.21, ns								

Fig	7-d subtype transition rate (%)									
									mean	s.e.m
4I	young	non to mush	0.00	0.33	0.28	0.22	0.73	0.07	0.27	0.10
		mush to non	0.59	0.43	0.85	0.45	0.94	0.00	0.54	0.13
	middle-aged	non to mush	0.15	0.26	0.47	0.50			0.35	0.07
		mush to non	0.15	0.09	0.59	0.00			0.21	0.11
	Paired Student's t-test within each group									
			P = 0.052, ns		P = 0.38, ns					
	Student's t-test between group									
		non to mush	P = 0.63, ns							
		mush to non	P = 0.13, ns							
	Note: the dominator is the sum of all spines									

Fig	% of mushroom spine transited to non-mushroom spine							
							mean	s.e.m
4J	young	18.18	20.00	21.05	22.50	14.81	0.00	16.09	3.10
	middle-aged	1.47	1.07	12.66	0.00			3.80	2.57
	young vs. middle-aged, Student's t-test								
	P = 0.035, *								
	Note: the dominator is the sum mushroom spines								

Supplementary Table 4. Summary of statistical analyses

Fig	Numeric data and statistical p value				
	ISI range (μm)	Probability density			
		y3	y5	y13	y17
	0-1	0.0717	0.0789	0.0438	0.0346
	1-2	0.1548	0.1502	0.1089	0.0787
	2-3	0.1729	0.1574	0.1105	0.088
	3-4	0.1535	0.1256	0.1045	0.1114
	4-5	0.1106	0.1015	0.0961	0.0974
	5-6	0.0938	0.0861	0.0896	0.0843
	6-7	0.059	0.0733	0.0788	0.0768
	7-8	0.0529	0.061	0.0667	0.073
	8-9	0.0389	0.0354	0.0543	0.0506
	9-10	0.0275	0.0379	0.0486	0.0515
	10-11	0.0154	0.0246	0.0398	0.0478
	11-12	0.0114	0.0133	0.0378	0.0421
	12-13	0.0101	0.0164	0.0233	0.0328
	13-14	0.008	0.0103	0.0181	0.0253
**5A**	14-15	0.0047	0.0067	0.0161	0.0159
	15-16	0.0047	0.0026	0.0105	0.0159
	16-17	0.0027	0.0056	0.0125	0.0159
	17-18	0.0013	0.0041	0.0084	0.0084
	18-19	0.0013	0.0005	0.008	0.0047
	19-20	0.0013	0.0015	0.0072	0.0075
	20-21	0	0.001	0.0032	0.0103
	21-22	0.0013	0.0021	0.0016	0.0056
	22-23	0.0007	0.0005	0.002	0.0028
	23-24	0	0	0.0016	0.0028
	24-25	0.0013	0.0005	0.0008	0.0019
	25-26	0	0.0005	0.0016	0.0019
	26-27	0	0	0.0012	0.0019
	27-28	0	0	0	0.0037
	28-29	0	0.0005	0.0008	0
	29-30	0	0.0005	0.0008	0
	30-31	0	0	0	0

Supplementary Table 5. Summary of statistical analyses

Fig		Numeric data and statistical p value			
		ISI (um)		ISI <2 um (%)	
		mean	peak	random	Observed
**5B-C**	y3	4.57	2.47	36.85	21.91
	y5	4.86	2.07	35.11	24.14
	y13	6.50	3.07	27.63	15.02
	y17	7.38	3.80	24.79	11.18
	mean	5.83	2.85	31.09	18.06
	s.e.m	0.58	0.33	2.51	2.60
		Paired Student's t-test		Paired Student's t-test	
		P = 0.0031, **		P = 0.00057, ***	

Fig		spine number within 8 um		Paired Student's t-test
	spine number	random	observed	
**5D**	2	91.73 ± 1.38	92.72 ± 1.56	P = 0.0061 **
	3	67.65 ± 3.97	59.08 ± 5.53	P = 0.0009 ***
	4	38.99 ± 4.82	24.53 ± 5.30	P = 3.90e-04 ***
	5	18.28 ± 3.61	7.53 ± 2.34	P = 1.30e-04 ***
	6	7.18 ± 1.94	1.84 ± 0.67	P = 0.0042 **

Fig		y3			y5				y13		y17	
**5E-F**	mean ISI (μm)	3.55	5.22	3.75	4.64	4.22	6.30	6.09	6.31	7.15	7.46	7.42
	scale, η	3.47	5.41	3.92	5.07	4.84	6.76	6.59	6.73	7.28	7.54	8.20
	shape, β	1.68	1.66	1.59	1.53	1.53	1.31	1.51	1.42	1.46	1.52	1.50

Fig		mean ISI		mean ISI of eliminated spines	
		without formation	with formation	shuffled	observed
**5G-H**		3.445	6.527	3.326	3.229
		5.099	7.886	4.949	4.463
		3.537	5.940	3.546	3.264
		4.415	7.015	4.339	4.261
		3.782	5.902	3.765	3.502
		6.103	10.902	6.314	5.551
		5.998	10.100	5.615	3.756
		6.866	11.786	6.443	5.491
		5.988	10.333	5.579	5.119
		7.041	12.786	6.885	6.332
		6.881	12.337	6.622	7.367
	mean	5.378	9.228	8.577	5.149
	s.e.m	0.398	0.758	0.974	0.569
		Paried Student's t-test			
		Without vs with	P = 1.87E-06, ***	shuffled vs. observed	P = 0.039, *

Supplementary Table 6. Summary of statistical analyses

Fig	Numeric data and statistical p value			
	ISI range (μm)	probability density		
		all	formed	eliminated
5I	0-1	0.0638	0.0839	0.0782
	1-2	0.1352	0.154	0.1544
	2-3	0.1416	0.1635	0.1648
	3-4	0.1267	0.1401	0.1342
	4-5	0.1049	0.1044	0.1068
	5-6	0.0878	0.0912	0.0762
	6-7	0.069	0.0569	0.0632
	7-8	0.0618	0.0562	0.0541
	8-9	0.0431	0.0292	0.0339
	9-10	0.0387	0.0394	0.0332
	10-11	0.0285	0.0182	0.0306
	11-12	0.0246	0.019	0.0189
	12-13	0.0169	0.0102	0.0104
	13-14	0.012	0.008	0.0085
	14-15	0.0097	0.008	0.0059
	15-16	0.0063	0.0044	0.0065
	16-17	0.0069	0.0022	0.0046
	17-18	0.0043	0.0022	0.0046
	18-19	0.0032	0.0022	0.002
	19-20	0.0037	0.0015	0.0026
	20-21	0.0026	0.0015	0.0013
	21-22	0.0015	0	0.002
	22-23	0.0013	0.0007	0.0007
	23-24	0.0007	0	0.0007
	24-25	0.001	0	0

Supplementary Table 7. Summary of statistical analyses

Fig	Numeric data and statistical p value				
supp 2D		d0-d7	d7-d14	d0-d7	d7-d14
	turnover rate (%)	formation	formation	elimination	elimination
		5.11	6.36	8.76	4.55
		7.81	6.13	5.86	5.17
		6.76	4.07	7.09	7.80
		7.45	5.09	5.42	5.31
		8.98	6.85	8.35	8.09
		13.32	8.37	5.42	7.53
		5.39	3.22	5.97	6.44
		1.85	1.23	1.24	3.23
		7.94	4.68	4.32	5.70
		7.15	8.37	7.74	7.43
		9.18	7.87	5.44	6.23
	mean	7.36	5.66	5.96	6.13
	s.e.m	0.82	0.65	0.60	0.44
		d0-d7 vs. d7-d14		formation	elimination
	Student's t-test			P = 0.14, ns	P = 0.83, ns

Fig		formation rate (%)	elimination rate (%)	mean formation	mean elimination
supp 2E	y3	5.72	6.69	6.03	6.55
		6.96	5.51		
		5.41	7.45		
	y5	6.26	5.36	7.31	6.58
		7.91	8.22		
		10.75	6.51		
		4.31	6.20		
	y13	1.54	2.23	3.91	3.63
		6.28	5.02		
	y17	7.76	7.58	8.14	6.71
		8.51	5.84		

Fig	turnover rate /100μm	young		middle-aged	
supp 2F		formation	elimination	formation	elimination
		3.32	3.88	0.55	0.80
		3.77	2.99	2.08	1.66
		2.18	2.99	2.21	2.16
		3.52	3.02	2.49	1.71
		3.60	3.74		
		5.57	3.37		
		1.42	2.05		
	mean	3.34	3.15	1.83	1.58
	s.e.m	0.46	0.21	0.38	0.25
	Paired Student's t-test within each group				
		P = 0.65, ns		P = 0.35, ns	
	young vs. middle-aged, Student's t-test				
	formation	P = 0.072, ns			
	elimination	P = 0.0023, **			

Supplementary Table 8. Summary of statistical analyses

Fig | **Numeric data and statistical p value**

supp 3E

2-wk mushroom spine turnover rate (%)											mean	s.e.m
formation	0	0	0	0	0	0	0	1.07	0	0	0.11	0.11
elimination	4.55	1.33	0	0	0	0	0.74	0	0	0	0.66	0.45

formation vs. elimination, Paired Student's t-test P=0.28, ns

4 months mushroom spine turnover rate	
formation	5/287
elimination	7/287

supp 4A

	y3	y5	y13	y17
mean ISI (μm)	4.57	4.86	6.50	7.38
scale, η	4.61	4996	6.84	7.66

supp 4C

ISI range (μm)	cumulative probability density							
	y3		y5		y13		y17	
	without	with	without	with	without	with	without	with
0-1	0.0751	0	0.0907	0.0045	0.0467	0	0.0383	0
1-2	0.2397	0.0076	0.2621	0.0452	0.1583	0.0152	0.1213	0.0221
2-3	0.4129	0.1374	0.4253	0.1312	0.2802	0.0254	0.2258	0.0294
3-4	0.5724	0.229	0.5518	0.2534	0.3918	0.0457	0.3432	0.0515
4-5	0.6887	0.3053	0.6557	0.3439	0.4921	0.1168	0.4452	0.0809
5-6	0.7795	0.4122	0.7355	0.4434	0.5836	0.2183	0.5351	0.1176
6-7	0.8348	0.4885	0.8035	0.5475	0.6576	0.2944	0.6133	0.2059
7-8	0.8894	0.5725	0.8569	0.6606	0.7255	0.3553	0.6865	0.2647
8-9	0.9252	0.6641	0.889	0.7149	0.7781	0.4518	0.7376	0.3015
9-10	0.9479	0.8168	0.9237	0.7873	0.8267	0.5279	0.7824	0.4191
10-11	0.9623	0.855	0.9438	0.8281	0.8634	0.5939	0.8267	0.4706
11-12	0.9719	0.8931	0.9589	0.8507	0.8984	0.6599	0.8649	0.5368
12-13	0.9783	0.9389	0.9743	0.8824	0.9183	0.7056	0.892	0.6029
13-14	0.9843	0.9542	0.9802	0.9321	0.9347	0.7614	0.9131	0.6618
14-15	0.9879	0.9695	0.9844	0.9548	0.9499	0.7919	0.929	0.7206
15-16	0.9901	0.9924	0.9861	0.9593	0.9589	0.8173	0.9428	0.7279
16-17	0.993	0.9924	0.9894	0.9774	0.9684	0.8477	0.9557	0.8235
17-18	0.9946	0.9924	0.9917	0.9819	0.9741	0.8934	0.9639	0.8529
18-19	0.9952	1	0.9927	0.9864	0.9803	0.9239	0.9677	0.8603
19-20	0.9965	1	0.9941	0.9864	0.9858	0.9492	0.9746	0.875
20-21	0.9965	1	0.9953	0.9864	0.9888	0.9543	0.9824	0.8971
21-22	0.9974	1	0.9965	0.9955	0.9905	0.9594	0.9845	0.9265
22-23	0.9981	1	0.9969	1	0.9926	0.9695	0.9862	0.9338
23-24	0.9984	1	0.9969	1	0.9936	0.9797	0.9884	0.9412
24-25	0.9997	1	0.9976	1	0.9941	0.9848	0.9905	0.9412

Supplementary Table 9. Summary of statistical analyses

Fig	ISI range (µm)	y3 All	y3 formed	y3 eliminated	y5 All	y5 formed	y5 eliminated	y13 All	y13 formed	y13 eliminated	y17 All	y17 formed	y17 eliminated
		probability density											
	0-1	0.0751	0.1065	0.1032	0.0907	0.1055	0.0935	0.0467	0.0518	0.0671	0.0383	0.0467	0.034
	1-2	0.1646	0.2199	0.2037	0.1714	0.2426	0.1761	0.1116	0.08	0.1204	0.083	0.07	0.1019
	2-3	0.1732	0.1443	0.1825	0.1632	0.1667	0.1761	0.1218	0.1671	0.1667	0.1045	0.1433	0.117
	3-4	0.1595	0.1581	0.127	0.1266	0.1308	0.137	0.1116	0.16	0.1481	0.1174	0.1233	0.117
	4-5	0.1163	0.1409	0.1005	0.1039	0.1034	0.1087	0.1003	0.1106	0.1019	0.1019	0.1067	0.1208
	5-6	0.0908	0.0619	0.082	0.0798	0.0591	0.0609	0.0916	0.1153	0.0856	0.0899	0.0967	0.0792
	6-7	0.0553	0.0515	0.0423	0.068	0.057	0.0739	0.074	0.0424	0.0648	0.0783	0.0833	0.0717
	7-8	0.0547	0.0584	0.0582	0.0534	0.0316	0.05	0.0679	0.0635	0.0394	0.0731	0.0733	0.0792
	8-9	0.0358	0.0206	0.0397	0.0321	0.0232	0.0261	0.0526	0.0353	0.0394	0.0512	0.0567	0.0302
	9-10	0.0227	0.0172	0.0185	0.0347	0.0316	0.0239	0.0486	0.0588	0.0486	0.0447	0.0467	0.0453
**supp 4D left**	10-11	0.0144	0.0069	0.0132	0.0201	0.0105	0.0217	0.0367	0.0235	0.037	0.0443	0.04	0.0604
	11-12	0.0096	0.0069	0.0079	0.0151	0.0127	0.0196	0.035	0.0282	0.0231	0.0383	0.02	0.0264
	12-13	0.0064	0.0034	0.0053	0.0153	0.0148	0.0109	0.0199	0.0165	0.0116	0.0271	0.01	0.0151
	13-14	0.0061	0.0034	0.0053	0.0059	0.0042	0.0022	0.0165	0.0118	0.0116	0.0211	0.0133	0.0189
	14-15	0.0035	0	0	0.0043	0	0	0.0151	0.0141	0.0116	0.0159	0.0133	0.0151
	15-16	0.0022	0	0	0.0017	0	0.0065	0.0091	0.0071	0.0046	0.0138	0.0033	0.0189
	16-17	0.0029	0	0.0026	0.0033	0	0	0.0095	0.0047	0.0093	0.0129	0.01	0.0075
	17-18	0.0016	0	0.0026	0.0024	0	0.0065	0.0057	0	0.0023	0.0082	0.01	0.0075
	18-19	0.0006	0	0	0.0009	0.0021	0.0022	0.0062	0	0	0.0039	0.0033	0.0075
	19-20	0.0013	0	0.0026	0.0014	0.0021	0	0.0055	0	0.0046	0.0069	0.0067	0.0038
	20-21	0	0	0	0.0012	0	0	0.003	0.0024	0.0023	0.0077	0.0033	0.0038
	21-22	0.001	0	0.0026	0.0012	0	0	0.0017	0	0	0.0022	0	0.0075
	22-23	0.0006	0	0	0.0005	0	0.0022	0.0021	0.0024	0	0.0017	0	0
	23-24	0.0003	0	0	0	0	0	0.0009	0	0	0.0022	0.0033	0.0038
	24-25	0.0013	0	0	0.0007	0	0	0.0006	0	0	0.0022	0	0

Fig	scale, η	All	Formed	Eliminated	Paired Student's t-test vs.	Formed	Eliminated
	y3	4.431	3.995	4.088	All	P = 0.0097, **	P = 0.085, ns
	y5	4.709	3.740	4.415	Formed		P = 0.34, ns
	y13	6.536	5.455	5.215			
	y17	7.122	6.289	6.639			
	mean	5.700	4.870	5.089			
**supp 4D right**	s.e.m	0.576	0.524	0.492			

Fig	shape, β	All	Formed	Eliminated	Paired Student's t-test vs.	Formed	Eliminated
**supp 4D right**	y3	1.604	1.509	1.457	All	P = 0.37, ns	P = 0.71, ns
	y5	1.428	1.486	1.467	Formed		P = 0.064, ns
	y13	1.471	1.656	1.512			
	y17	1.529	1.623	1.523			
	mean	1.508	1.569	1.490			
	s.e.m	0.033	0.036	0.014			
