# Supplementary material for: Stability and dynamics of dendritic spines in macaque prefrontal cortex
Source: Natl Sci Rev. 2022 Jun 27;9(9):nwac125. doi: 10.1093/nsr/nwac125 (PMC9521340; doi:10.1093/nsr/nwac125)
Supplement: nwac125_Supplemental_Files [file nwac125_supplemental_files.zip › Supplementary_data-Meterials_and_Methods.docx]

**METERALS AND METHODS**

**Animals**

Four male rhesus monkeys (*Macaca mulatta*; 3-year-old, 5 kg, y3; 5-year-old, 7 kg, y5; 13-year-old, 9 kg, y13; and 17-year-old, 8 kg, y17) participated in this study. All procedures were approved by the Animal Care and Use Committee of the Institute of Neuroscience, Chinese Academy of Sciences, Shanghai, China.

**MRI acquisitions**

All monkeys were imaged using Siemens Tim Trio 3T MRI (Siemens, Germany) to acquire whole brain T1 images (voxel size: 0.5 mm×0.5 mm×0.5 mm) for localization of the principal sulcus and the arcuate sulcus, and estimation of the gray matter thickness of dlPFC. The information was used to determine the location and depth of viral injection.

**Surgery, virus injection and optical window implantation**

During the surgery, monkeys were under general anesthesia in strictly sterile conditions with vital signs monitoring include SpO_2_, ECG, EtCO_2_ and body temperature, and were applied with intraoperative antibiotics. Anesthesia was induced by *Zoletil* (Tiletamine hydrochloride and zolazepam hydrochloride, 5 mg/kg, i.m.) and maintained using 1.7~2.0% isoflurane in oxygen. We first implanted a screw-cement base on the skull, consisting of acrylic bone cement (TSMRI, China) and 6-7 custom-made titanium T-head bolts and nuts surrounding the presumptive imaging field, to help seal the later implanted imaging cranial window. One month after the base implantation, a craniotomy in dlPFC (~21 mm in diameter) and durotomy were performed to expose the cortex. A 1:9 mixture of AAV2/8-hSyn-Cre (titer: 1×10^13^ VG/ml, diluted 300~1000-fold in PBS) and AAV2/9-CAG-FLEX-EGFP (titer: 4×10^13^ VG/ml) was injected to sparsely label L5 pyramidal neurons of dlPFC. At least 5 injections were made for each monkey with 2-3 mm lateral separations, each injection with 100~300 μl virus mixture at depths of 1.4-1.8 mm, determined by the MRI images. Then a custom-made glass window (diameter 20 mm, thickness 0.3 mm) with a PMMA ring (adhered with NOA81, Norland, Inc., USA) was used to cover the cortex after virus injection (Supplementary Fig. 1A). Bone wax (W810T, Ethicon, USA) and acrylic bone cement were used to seal the gap between the imaging window and the skull (Figure. 1C). Head-posts were installed on the screw-cement base parallel to the plane of imaging window as much as possible and allow sufficient working space for the objectives of the two-photon microscope. Ceftriaxone (50 mg/kg, i.m.) and dexamethasone (2 mg/kg-0.25 mg/kg gradually decreasing, i.m.) were injected twice a day for one week after surgery.

**Two-photon microscopy**

Monkeys were anaesthetized with isoflurane (1.7-2%) and fixated using the implanted head-posts. Image stacks were taken every 1 μm, from the cortical surface to 60-100 μm deep, with a two-photon microscope (Bergamo II, Thorlabs, USA) and ThorImage software (Thorlabs, USA), at a frame rate of 30 Hz, 20 or 25 frames acquired for each depth. The objectives used were 16×, 0.8 numerical aperture (Nikon, Japan) and 25×, 1.05 numerical aperture (Olympus, Japan). A digital zoom of 6.4 (16× objective) or 4 (25× objective) was used, with pixel sizes of 0.24 μm ×0.24 μm (16× objective) or 0.22 μm×0.22 μm (25× objective). A Ti:sapphire laser (Mai Tai DeepSee, Spectra Physics, USA) was used as the light source, and tuned to 910 nm for imaging GFP signals. Using the surface vasculature and dendritic arbors as references, we achieved repeated imaging of same 2-4 ROIs (virus injection sites) for all monkeys. For optimal imaging quality, laser beam from the objective was adjusted to near perpendicular (less than 2°) to the optical window by rotating the microscope.

**Histology and immunofluorescence imaging**

The expression profile of virus injection was analyzed on monkey y13. The animal was given a lethal dose of pentobarbital sodium (100 mg/kg, i.v.) and anticoagulant heparin (100 U/kg, i.v.), the descending aorta clamped and perfused transcardially with 0.1M phosphate-buffered saline (PBS, pH 7.4) for 10 min (0.3 L/Kg) and then 4% paraformaldehyde in PBS for 10 min (0.3 L/Kg). Following perfusion, the brain was removed and post-fixed in 4% paraformaldehyde for 48 h at 4^o^C. A block of dlPFC (10 mm×10 mm×5 mm) was dissected from the brain along the arcuate sulcus, and cut on a vibratome in 100-μm coronal sections. The brain sections were blocked with 10% normal donkey serum (NDS), 1% Triton-X 100 and 5% BSA for 1 h at room temperature. After blocking, the brain sections were incubated with mouse anti-NeuN (1:300, Millipore, cat# MAB377) over night at 4^o^C, rinsed with PBS for three times and incubated with secondary antibody Alexa Fluor® 594 (1:600, ThermoFisher Scientific, cat#A-21203) in dark for 1-2 h at room temperature. Then the brain sections were counter-stained with DAPI (Millipore, cat# D9542) in dark for 20 min, rinsed with PBS for three times and mounted with fluorescence mounting medium (DAKO). Immuno-fluorescence images were acquired using Olympus VS120 (Japan) with a 10× objective (Supplementary Fig. 1B).

**Data analysis**

**Image alignment**

The heart beat and breathing of monkeys causes significant vertical shifting of the focal plane (up to 5 μm, 0.5-2 Hz), which can induce severe blurring and z-shifting of imaged structure in consecutive image frames. To solve this problem, we used t-distributed stochastic neighbor embedding (t-sne) to rearrange the sequences of the image stacks in order to smoothen image transitions between adjacent frames. First, we binned the stack frames to 6-7.5 Hz after x-y alignment using TurboReg (ImageJ plugin), then we computed the correlation coefficient of each frame with its nearest 150 neighboring frames in both directions (for each frame, we searched from 30 μm below to 30 μm above the focal plane for alignment) in the binned stacks. The 2-D correlation coefficient was sorted by t-sne to maximize the distance of the first and last frames and minimize the differences of adjacent frames in the binned stacks (Supplementary Fig. 2A). The new image stacks were generated by the sorted indices and averaged for each imaging depth after x-y alignment.

**Spine density and inter-spine interval measurements**

Inter spine intervals (ISIs) were measured using the semi-automatic ImageJ plugin, Simple Neurite Tracer (Supplementary Fig. 2C), in 3D reconstructed images. The summation of ISIs on one dendritic segment was used as the length of this dendritic segment. Spine density is calculated as spine count divided by dendritic segment length for each dendritic segment (Fig. 2B). Dendritic segments with imaged length of >50 μm were included in the spine density analyses.

To ensure accuracy in ISI measurement and consistency of dendritic segment lengths among image sessions, each ISI in the first imaging session was determined by taking an average of three separate measurements; in the following imaging sessions, those ISIs between persistent spine pairs were inherited from the previous session, and the additional ISIs resulted from spine formation and elimination were calculated as follows (see Supplementary Fig. 2C): the ISIs for a newly formed spine are the two ISIs between the newly formed spine and its two pre-existing neighbors; the ISI for an eliminated spine is the summation of the two ISIs between eliminated spine and its two adjacent persistent neighbors.

**Spine turnover and survival**

Images of the same dendritic segments (t0 vs. t0 + 4h, d0 vs. d7, and d7 vs. d14, in Fig. 2) were all opened using ImageJ for determining spine formation and elimination. The percentages of spine formation and elimination were calculated by comparing the number of spine formation and elimination obtained on d7 and d14 with the number of spine on d0 and d7 (Δd7 + Δd14)/(d0 + d7) for each ROI (virus injection site), giving the similar spine formation and elimination rates between d0-d7 and d7-d14 (Supplementary Fig. 2D). Spine formation and elimination rates against dendritic segment length were also calculated (Supplementary Fig. 2F). Percentages of 7-day and 14-day survived spine for all spines were determined by comparing the number of persistent spines from d0 to d7 and d14 to the number of initial spine population on d0, respectively. To compare the elimination rates of all spines and newly formed spines, we calculated the percentages of eliminated spine during d7-d14 for all spines observed on d7, and eliminated new spines during d7-d14 for all new spines formed during d0-d7. We also calculated the percentages of all new spines formed during d0-d7 for all spines observed on d7 and eliminated new spines for all eliminated spines during d7-d14.

**Long-term survival rate**

To compare the long-term survival rates of all spines and newly formed spines, we repeatedly imaged the same dendritic segments for at least 5 image sessions in 7, 14 or 107-day intervals in monkeys y3, y5, and y13. We calculated the survival rates of the spines, observed in the every first image session, in the following image sessions and those of newly formed spine between image sessions 1-2 and image sessions 2-3. The survival rates ($sr$) of newly formed spines against observed dates were fitted with an exponential function：

$$sr=\left( 1-p \right)* e^{\left( \frac{-date}{\tau} \right)}+p$$

Where $p$ is the plateau value (stabilized rate) and τ is the elimination constant of new spines.

**Repeated turnover analysis**

We compared the observed number of turnover events in all dendritic spine sites with those predicted by assuming random turnover (simulated based on observed average turnover rates) in 6 image sessions. The averaged turnover rate was calculated as the average of formation and elimination rates for all imaging sessions. The observed and simulated turnover events of 4 or more were very rare, and were therefore included in the **>=** 3 turnover group.

**Categorization and quantification of morphological changes**

By virtue of sparse labeling and high image quality, spines can be categorized into three subtypes based on morphology[1-3]. Mushroom spines were identified as spines with head diameter/neck diameter >2-fold, or head diameter/nearby dendritic shaft diameter >1.2-fold (Supplementary Fig. 3A). Filopodia were identified as long thin filiform structures with length >4 μm, or structures with maximum fluorescent value <1/3 of nearby dendritic segments (Supplementary Fig. 3B). The remaining structures were classified as non-mushroom spines because they constitute a continuous population based on the measurements of spine head and spine neck.

For non-mushroom spines, reshaping events were recorded with spine length increased or decreased by >15% (Supplementary Fig. 3C). Transitions from non-mushroom spine to mushroom spine were recorded with spine head diameter swelling >30%, and emergence of typical mushroom spine feature; transitions from mushroom spine into non-mushroom spine were recorded with head diameter shrinking >30%, and emergence of non-mushroom spine feature (Supplementary Fig. 3D). To reliably calculate the turn-over and subtype transition rates of mushroom spines, we used 10 out of 11 injection sites which labeled more than 10 mushroom spines in Fig. 4D, 4I and 4J.

**Fitting ISIs with Weibull distribution**

To find the best fitted Weibull distribution of the probability distribution (*p*) of ISIs (*x*) for all, newly formed, and eliminated spines:

$$p(x)=\frac{\beta}{\eta}\left(\frac{\text{x}}{\eta}\right)^{\beta-1}\exp^{-\left(\frac{x}{\eta}\right)^{\beta}}$$

we minimized the sum of squared residuals between observed and fitted data points with either gradient of scale (η) and shape (β) value of Weibull function with step size 0.01. For newly formed and eliminated spines, we fitted with pooled data from all monkeys (Fig. 5I) and data from single monkey (Supplementary Fig. 4D), and both showed similar ISI distributions of newly formed and eliminated spines. The peak ISIs of the observed average ISI distribution was determined as the ISI with the highest probability in the best fitted Weibull distribution.

To simulate the spine occurrence rates between 2 existing spines, we calculated the occurrence rates (*r*) of distances (*x*) from each of the 2 existing spines as:

$$r(x)=\frac{\beta}{\eta}\left(\frac{\text{x}}{\eta}\right)^{\beta-1}$$

and normalized the summation of occurrence rates along the interval to 1.

**Fraction of spine clustering within 8 μm**

To quantitatively measure the fractions of spine clustering within 8 μm, we calculated the percentages of observed and Poisson redistributed (λ = 1) spines clustered within 8 μm in all spines for each virus injection site. Clustered 2 to 6 spines within 8 μm were used for the comparisons. The percentages of observed and randomized clustered spines were compared with paired Student’s t-test.

**Correlation of spine density with formation and elimination**

We compared the accumulated percentage of formed and eliminated spine by sorted normalized spine density from low to high for all dendritic segments (>50 μm) for all monkeys. We measured the spine density and the number of spine formation/elimination for each dendritic segment, and then normalized the spine density by mean density of each ROI. We then pooled all segments from all ROIs and sorted them by the normalized density from low to high. The cumulative percentages of spine formation and elimination were calculated by the cumulative number of formed and eliminated spine in sorted segments divided by the total number of spine formation and elimination in all segments, respectively. The two cumulative percentages of spine formation and elimination in sorted dendritic segments were compared using Kolmogorov-Smirnov test.

**Statistics**

Student’s t-test and paired Student’s t-test were used in this article, data point can be virus injection site or single monkey according to context which has been illustrated in figure legends as well as in Supplementary Tables. Kolmogorov-Smirnov test was used for cumulative probability comparison in Fig. 5J, Supplementary Fig. 4C and 4E. All statistical analyses were performed using MATLAB2018a (Mathworks). For detailed information of statistical analyses, see Supplementary Tables.

# References

1. Zagrebelsky, M, Holz, A, Dechant, G*, et al.* The p75 neurotrophin receptor negatively modulates dendrite complexity and spine density in hippocampal neurons. *J Neurosci*. 2005; **25**(43): 9989-99.

2. Harris, KM, Jensen, FE, Tsao, B. Three-dimensional structure of dendritic spines and synapses in rat hippocampus (CA1) at postnatal day 15 and adult ages: implications for the maturation of synaptic physiology and long-term potentiation. *J Neurosci*. 1992; **12**(7): 2685-705.

3. Grutzendler, J, Kasthuri, N, Gan, WB. Long-term dendritic spine stability in the adult cortex. *Nature*. 2002; **420**(6917): 812-6.
